# Gut Microbial Signatures of Frailty and Functional Independence in Centenarians and Older Adults

**DOI:** 10.1093/geroni/igaf122.4410

**Published:** 2025-12-31

**Authors:** Meghan Short, Tanya Karagiannis, Ye Chen, Daniel Segrè, Sarah Bald, Stacy Andersen, Thomas Perls, Paola Sebastiani

**Affiliations:** Tufts University School of Medicine, Medford, Massachusetts, United States; Tufts Medical Center, Boston, Massachusetts, United States; Tufts Medical Center, Boston, Massachusetts, United States; Boston University, Boston, Massachusetts, United States; Boston University, Boston, Massachusetts, United States; Boston University, Boston, Massachusetts, United States; Boston University, Boston, Massachusetts, United States; Tufts Medical Center, Boston, Massachusetts, United States

## Abstract

Frailty and functional limitations decrease quality of life for older adults and are associated with myriad health risks. The gut microbiome is a potential therapeutic avenue for promoting functioning among older adults, but a clearer understanding of mechanisms by which commensal gut bacteria may affect frailty and functional independence is needed. In this study, we analyzed data from an ongoing cohort study, Integrative Longevity Omics (ILO), which has 418 shotgun metagenomics samples of the gut microbiome from centenarians and their offspring. We measured associations of microbial alpha/beta diversity and species with function measures (activities of daily living [ADLs] and instrumental activities of daily living [IADLs]) and Fried frailty phenotype domains (physical activity, fatigability, grip strength, weight loss). Species-level alpha diversity decreased with increasing Pittsburgh fatigability score(β=-0.0051, 95% CI:-0.0098 to -0.0003, p = 0.038), adjusting for age, sex, and education. Beta diversity (Bray-Curtis dissimilarity) was associated with ADLs(R2=0.005, p = 0.002), IADLs(R2=0.005, p = 0.006), and fatigability(R2=0.010, p ≤ 0.001), adjusting for age, sex, and education using PERMANOVA. Finally, we identified several species associated with multiple frailty and functioning scores, including Anareostipes hadrus, which was associated with improved ADLs, IADLs, fatigability, and lower likelihood of substantial weight loss, and is a known anti-inflammatory butyrate producer. Worse ADLs/IADLs and greater fatigability was associated with Clostridium innocuum, a commensal gut bacteria with documented potential for virulence. These results identify microbial species for further analysis in association with other ‘omics layers and for future functional studies to understand their potential mechanistic effects on frailty and functioning outcomes in older adults.

